# Foam Rolling Training Effects on Range of Motion: A Systematic Review and Meta-Analysis

**DOI:** 10.1007/s40279-022-01699-8

**Published:** 2022-05-26

**Authors:** Andreas Konrad, Masatoshi Nakamura, Markus Tilp, Olyvia Donti, David G. Behm

**Affiliations:** 1 Present Address: Institute of Human Movement Science, Sport and Health, Graz University, Mozartgasse 14, 8010 Graz, Austria; 2 Institute for Human Movement and Medical Sciences, Niigata University of Health and Welfare, Niigata, Japan; 3 Sports Performance Laboratory, School of Physical Education and Sport Science, National and Kapodistrian, University of Athens, Athens, Greece; 4 School of Human Kinetics and Recreation, Memorial University of Newfoundland, St. John’s, Newfoundland and Labrador Canada

## Abstract

**Background:**

A single foam-rolling exercise can acutely increase the range of motion (ROM) of a joint. However, to date the adaptational effects of foam-rolling training over several weeks on joint ROM are not well understood.

**Objective:**

The purpose of this meta-analysis was to investigate the effects of foam-rolling training interventions on joint ROM in healthy participants.

**Methods:**

Results were assessed from 11 studies (either controlled trials [CT] or randomized controlled trials [RCTs]) and 46 effect sizes by applying a random-effect meta-analysis. Moreover, by applying a mixed-effect model, we performed subgroup analyses, which included comparisons of the intervention duration (≤ 4 weeks vs > 4 weeks), comparisons between muscles tested (e.g., hamstrings vs quadriceps vs triceps surae), and study designs (RCT vs CT).

**Results:**

Our main analysis of 290 participants with a mean age of 23.9 (± 6.3 years) indicated a moderate effect of foam-rolling training on ROM increases in the experimental compared to the control group (ES = 0.823; *Z* = 3.237; 95% CI 0.325–1.322; *p* = 0.001; *I*^2^ = 72.76). Subgroup analyses revealed no significant differences between study designs (*p* = 0.36). However, a significant difference was observed in the intervention duration in favor of interventions > 4 weeks compared to ≤ 4 weeks for ROM increases (*p* = 0.049). Moreover, a further subgroup analysis showed significant differences between the muscles tested (*p* = 0.047) in the eligible studies. Foam rolling increased joint ROM when applied to hamstrings and quadriceps, while no improvement in ankle dorsiflexion was observed when foam rolling was applied to triceps surae.

**Conclusion:**

Longer duration interventions (> 4 weeks) are needed to induce ROM gains while there is evidence that responses are muscle or joint specific. Future research should examine possible mechanisms underpinning ROM increases following different foam-rolling protocols, to allow for informed recommendations in healthy and clinical populations.

## Key Points

**Table:** 

Our meta-analysis revealed that foam-rolling training interventions can increase joint ROM in young healthy participants.
When the muscles examined in the eligible studies were considered, it was found that joint ROM increases following foam-rolling training are muscle- or joint-specific.
A duration of more than four weeks of foam-rolling training should be applied to induce improvements in joint ROM.

## Introduction

In a warm-up setting, several interventions are performed to increase the range of motion (ROM) of a joint. Stretching is widely used throughout all sports and different populations [1–9]; however, during the last decade, foam rolling has also become a popular warm-up technique to increase joint ROM [1, 10–14]. A recent meta-analysis reported that foam rolling is similarly as effective as stretching for increasing joint ROM acutely [12]. Furthermore, although an acute bout of stretching with a long duration (i.e., ≥ 60 s per muscle group) in isolation (with no dynamic warm-up activities) may transiently decrease strength and power performance [2, 3, 8, 15], no subsequent performance deficits have been reported after an acute bout of foam rolling [16, 17].

Regarding long-term (i.e., chronic/training) interventions to increase joint ROM, a number of studies have reported that different types of stretching (e.g., static, ballistic, proprioceptive neuromuscular facilitation [PNF]) [18] are effective [19–25]. However, evidence on the training effects of foam rolling on joint ROM is limited and inconsistent. For example, Hodgson et al. [26] reported no increases in joint ROM following a 4-weeks intervention while Kiyono et al. [27] found a significant increase in ROM after 5 weeks of training.

Since recent research suggests that foam rolling is equally effective in inducing acute changes in ROM compared to stretching [12], there is a need to summarize all the available evidence and to conduct a meta-analysis on the longer term training effects of foam rolling on ROM. Hence, this systematic review and meta-analysis aims to examine if foam-rolling training interventions can increase joint ROM in healthy participants. Moreover, subgroup analyses will examine specific responses due to intervention duration (e.g., ≤ 4 weeks vs > 4 weeks), within various muscles (e.g., hamstrings vs quadriceps vs triceps surae), and study designs (randomized controlled trials [RCT] versus controlled trials [CTs]).

## Methods

This review was conducted according to the PRISMA guidelines and the suggestions from Moher et al. [28] for systematic reviews with meta-analysis.

### Search Strategy

An electronic literature search was performed in PubMed, Scopus, and Web of Science. Papers were considered if they were published up to 29th September 2021. The terms used to detect long-term foam-rolling intervention studies were similar to those used in a recent review on the long-term effects of stretching on ROM (i.e., chronic effects, training effects, effects, long-term, and intervention) [22]. Moreover, to find studies dealing with foam rolling, the search “terms foam rolling, self-myofascial release, roller massage, and foam roller” were used according to previous meta-analyses [17, 29]. To detect flexibility studies, the search terms “flexibility and range of motion” were used [22]. The search code for all three databases was (“chronic effects” OR “training effects” OR “effects” OR “long-term” OR “intervention”) AND (“foam rolling” OR “self-myofascial release” OR “roller massage” OR “foam roller”) AND (“flexibility” OR “range of motion”). The systematic search was carried out by two independent researchers (AK, MN). In the first step, all the hits were screened by their title and abstract. If the content of a study remained unclear, the full text was screened to identify the relevant papers. Following this independent screening process, the researchers compared their findings. Disagreements were resolved by jointly reassessing the studies against the eligibility criteria.

### Inclusion and Exclusion Criteria

This review considered studies that investigated the long-term training effects of foam rolling on joint ROM in healthy participants. We included peer-reviewed original studies including English and German languages. The studies were included when they were either RCTs or CTs with an intervention duration ≥ 2 weeks [22]. This implied that we excluded studies that were dealing with the acute effects of foam rolling (or interventions that were < 2 weeks’ duration), investigated any combined treatment (e.g. foam roller combined with stretching), or had another treatment as control condition (e.g., stretching). Moreover, we excluded review papers, case reports, special communications, letters to the editor, invited commentaries, conference papers, or theses.

### Extraction of the Data

From the included papers, the characteristics of the participants, the sample size, the study design, the characteristics of the intervention (i.e., weeks of intervention, frequency of intervention per week, duration of each training session per muscle tendon unit, pressure of the foam roller, frequency the foam roller) was applied and the results of the main variables (flexibility parameters) were extracted. For the flexibility parameters pre- and post-intervention values plus standard deviations of the foam rolling and control groups were extracted. If some of the required data were missing in the included studies, the authors of the studies were contacted via email or similar channels (e.g., ResearchGate).

### Statistics and Data Synthesis

The meta-analysis was performed using Comprehensive Meta-Analysis software, according to the recommendations of Borenstein et al. [30]. By applying a random-effect meta-analysis, we assessed the effect size in terms of the standardized mean difference. If any study reported more than one effect size, the mean of all the outcomes (effect sizes) within that study was used for the analysis and was defined as combined (as suggested by Borenstein et al. [30]). Moreover, by applying a mixed-effect model, we performed subgroup analyses. Although there is no general rule of thumb [30], we only performed subgroup analyses when there were ≥ 3 studies included in the respective subgroups. Consequently, we were unable to perform further subgroup analyses on activity level (highly active vs recreational) or sex. However, subgroup analyses for the weeks of intervention (≤ 4 weeks vs > 4 weeks), the muscles tested (hamstrings, quadriceps, triceps surae, and the rest of the muscles), and the study design (RCTs vs CTs) were performed. We have chosen 4 weeks as a cut-off since it is a typical duration in stretching studies and it was half of the longest intervention duration of the eligible studies (i.e., 8 weeks). In the case that a muscle or muscle group was examined in fewer than three studies (i.e., rectus femoris, infraspinatus, adductors), the findings were summarized into a subgroup named “rest of the muscles”. To determine differences between the effect sizes of the subgroups, Q-statistics were applied [30]. According to the recommendations of Hopkins et al. [31], the effects for a standardized mean difference of < 0.2, 0.2–0.6, 0.6–1.2, 1.2–2.0, 2.0–4.0, and > 4.0 were defined as trivial, small, moderate, large, very large, and extremely large, respectively. *I*^2^ statistics were calculated to assess the heterogeneity among the included studies, and thresholds of 25%, 50%, and 75% were defined as having a low, moderate, and high level of heterogeneity, respectively [32, 33]. An alpha level of 0.05 was defined for the statistical significance of all the tests.

### Risk of Bias Assessment and Methodological Quality

The methodological quality of the included studies was assessed using the Physiotherapy Evidence Database (PEDro) scale. In total, 11 methodological criteria were rated by 2 independent researchers (AK, MN) and were assigned either one or no point. Hence, higher scores indicated better methodological quality of the study. In the case of conflict between the two researchers, the methodological criteria were reassessed and discussed. Moreover, statistics of the Egger’s regression intercept test and visual inspection of the funnel plot were applied to detect possible publication bias.

## Results

### Results of the Search

**Fig. 1:**
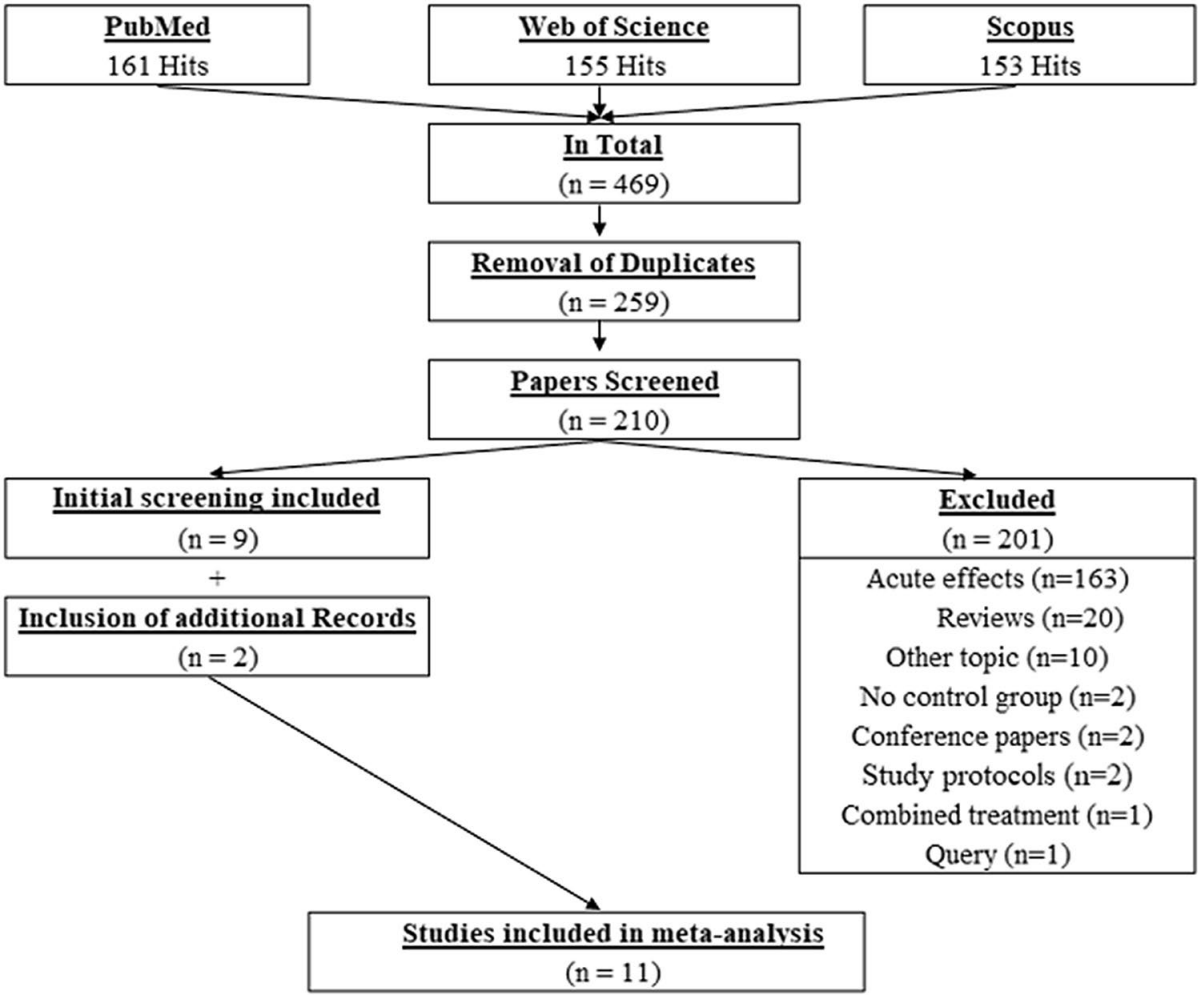
PRISMA flowchart

Overall, after removal of the duplicates, 210 papers were screened, from which 9 papers were found to be eligible for this review. However, following the additional search of the references (search through the reference list) and citations (search through Google Scholar) of the 9 already included papers, two more papers were identified as relevant. Therefore, in total, 11 papers were included in this systematic review and meta-analysis. The search process is illustrated in Fig. 1.

Overall, 46 effect sizes could be extracted from these studies. In summary, 290 participants with a mean age of 23.9 (± 6.3 years) participated in the included studies. Table 1 presents the characteristics and outcomes of the 11 studies.

**Table 1:** Characteristics of the included studies (*n* = 11)

Study	Participants	Intervention duration (weeks)	Training per week	Application (s) per training and muscle group	Total load (s)/muscle group	Frequency of Foam Rolling (one direction) (s)	Pressure of roller	RCT or CT	Outcome
Boguszewski et al. [65]	*N* = 37 physically active women; IG: *n* = 19 (age = 22.8 ± 2.3), CG: *n* = 18 (age 24.4 ± 1.6)	8	2	nr	nr	nr	nr	RCT	Sit and reach (bl)
Guillott et al. [37]	*N* = 30 professional national-level male Rugby players (age 18.85 ± 1.1)	7	~ 2,1	20 or 40	300 or 600	1.5	carefully applied pressure	RCT	Side split, Active straight leg (bl), Active flexed leg raising of the hip (bl), Active hip extension (bl), Active knee extension(bl), Active dorsiflexion (bl)
Hodgson et al. [26]	*N* = 23 recreational active; 13 males (25.1 ± 2.9); 10 females (age 24.9 ± 4.3)	4	3	120	1440 or 2880	1	7/10 VAS	RCT	Hamstrings Active ROM, Hamstrings Passive ROM, Quadriceps Active ROM, Quadriceps Passive ROM
Junker and Stöggl [55]	*N* = 26 recreational active male; IG: *n* = 13 (age 31.0 ± 8.5), CG: *n* = 13 (age 30.0 ± 9.0)	4	3	105	1260	1.75	Pain threshold	RCT	Stand and reach
Junker and Stöggl [58]	*N* = 26 recreational active male and female; IG: *n* = 14 (age 30.5 ± 10.2), CG: *n* = 12 (age 29.1 ± 6.9)	8	2	95	1520	nr	Mild to moderate pain (7/10 on VAS)	RCT	Sit and reach
Kiyono et al. [27]	*N* = 30 non-athlete male and female (age 20.8 ± 0.6)	5	3	90	1350	1	7/10 VAS	RCT	Dorsiflexion ROM
Le Gal et al. [45]	*N* = 11 advanced level male and female tennis players (age 15 ± 3)	5	3	180	2700	nr	Under the threshold of pain	CT	Glenohumeral internal ROM
Li et al. [56]	*N* = 40 physically active male and female with knee pain IG: *n* = 20 (age 38.16 ± 2.39), CG: *n* = 20 (age 36.33 ± 2.54)	8	1,4	nr	nr	nr	Until pain tolerance	RCT	Knee flexion ROM
Miller and Rockey [66]	*N* = 23 male and female college students with hamstrings tightness (20.53 ± 3.71)	8	3	180	4320	nr	nr	RCT	Active knee extension (bl)
Sandrey et al. [46]	*N* = 10 moderately active male and female with restrictions on knee extension and or flexion ROM (age 21.1 ± 2.0)	3	2	120	720	nr	Pressure with little discomfort	CT	Knee flexion and extension ROM
Stovern et al. [38]	*N* = 34 recreationally active male and female; IG: *n* = 20 (age 20.8 ± 1.70), CG: *n* = (age 20.8 ± 1.19)	6	3	60	1080	nr	nr	CT	Dorsiflexion and knee flexion ROM, Sit and reach

*bl* bilateral, *CG* control group, *CT* controlled trial, *IG* intervention group, *nr* not reported, *RCT* randomized controlled trial, *ROM* range of motion, *VAS* visual analogue scale

### Risk of Bias Assessment and Methodological Quality

**Fig. 2:**
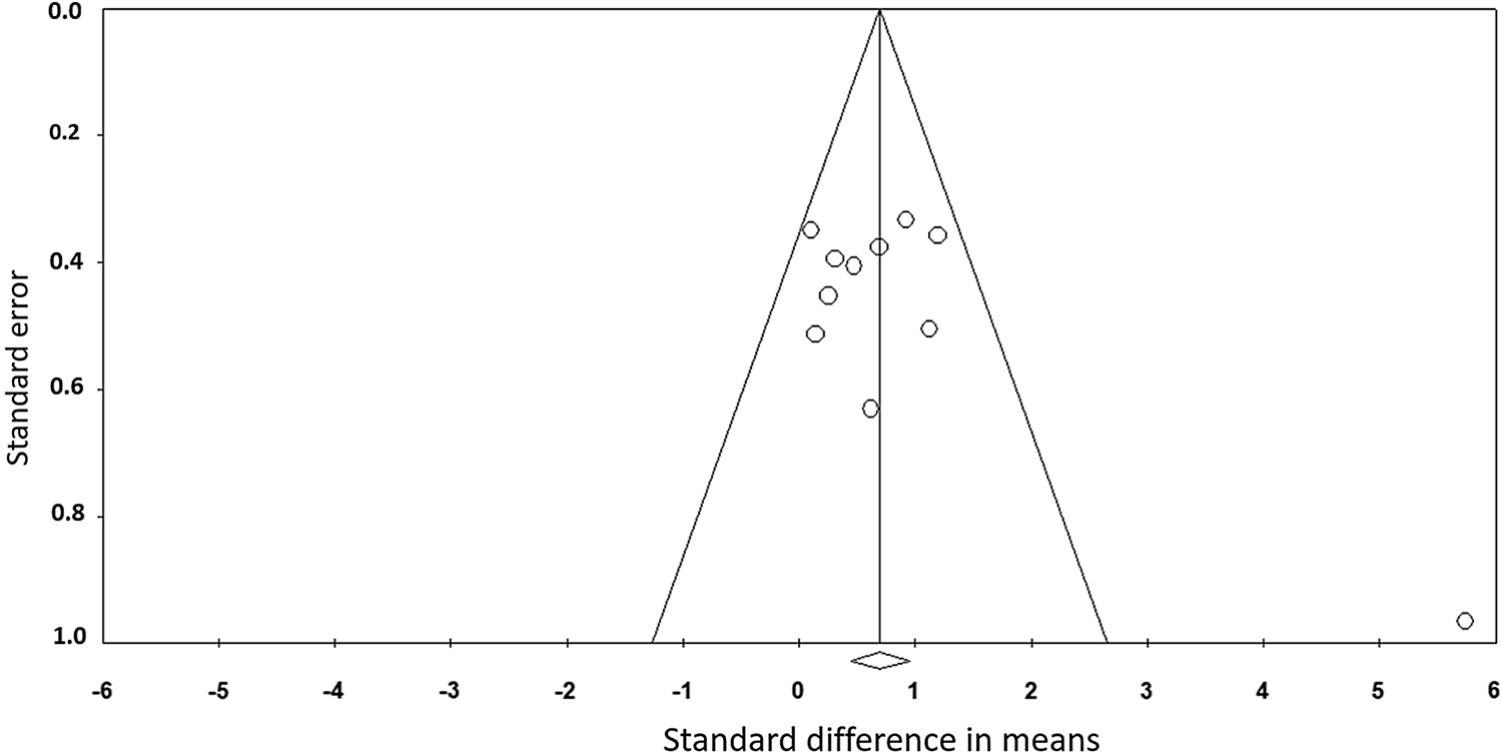
Funnel plot analysis

Figure 2 shows the funnel plot, including all 11 studies in this meta-analysis. A visual inspection of the funnel plot and the Egger’s regression intercept test (intercept 4.26; *p* = 0.07) indicated a tendency of reporting bias. The methodological quality, as assessed with the PEDro scale, revealed a range of scores between 5 and 8 points (out of 10) for all the included studies. The average PEDro score value was 6.36 (± 0.92), indicating a low risk of bias [34, 35]. The two assessors agreed with 93.4% of the 121 criteria (11 studies × 11 scores). The mismatched outcomes were discussed, and the assessors agreed on the scores presented in Table 2.

**Table 2:** PEDro scale of the included studies

Study	Inclusion criteria^a^	Random allocation	Concealed allocation	Similarity at baseline	Subject blinding	Therapist blinding	Assessor blinding	> 85% follow-up	Intention-to-treat analysis	Between-group comparison	Point estimates and variability	Total
Boguszewski et al. [65]	1	1	0	1	0	0	0	1	1	1	1	6
Guillott et al. [37]	1	1	0	1	1	0	1	1	1	1	1	8
Hodgson et al. [26]	1	1	0	1	0	0	0	1	1	1	1	6
Junker and Stöggl [55]	1	1	0	1	0	0	0	1	1	1	1	6
Junker and Stöggl [58]	1	1	0	1	0	0	0	1	1	1	1	6
Kiyono et al. [27]	1	1	0	1	0	0	0	1	1	1	1	6
Le Gal et al. [45]	1	1	0	1	0	0	1	1	1	1	1	7
Li et al. [56]	1	1	1	1	0	0	1	1	1	1	1	8
Miller and Rockey [66]	1	1	0	1	0	0	0	1	1	1	1	6
Sandrey et al. [46]	1	1	0	1	0	0	0	1	1	1	1	6
Stovern et al. [38]	1	0	0	1	0	0	0	1	1	1	1	5

*PEDro* Physiotherapy Evidence Database

^a^Was not counted for the final score; 1 = one point awarded; 0 = no points awarded

### Overall Effects

**Fig. 3:**
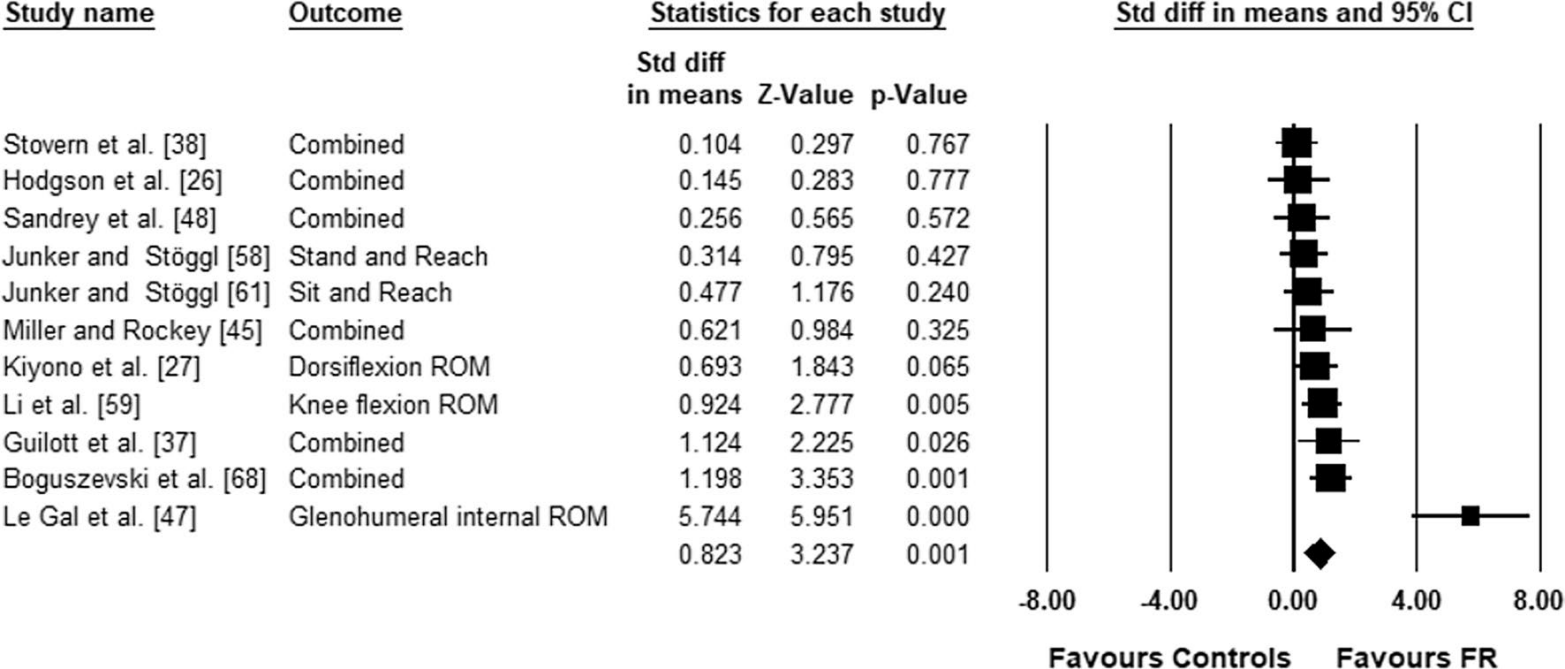
Forest plot presenting the 11 included studies investigating the effects of FR on ROM *CI* confidence interval, combined mean of the selected outcomes of one study, *FR* foam rolling, *ROM* range of motion, *Std diff* standardized difference

The meta-analysis on joint ROM revealed a moderate effect size in favor of foam rolling compared to the control condition (ES = 0.823; *Z* = 3.237; 95% CI 0.325–1.322; *p* = 0.001; *I*^2^ = 72.76). Figure 3 presents the forest plot of the meta-analysis, sorted by the standard difference in means beginning with the lowest value (0.104) up to the highest value (5.744).

### Subgroup Analysis

A summary of all the subgroup analyses is provided in Table 3. The subgroups analyzed were the weeks of intervention (≤ 4 weeks vs > 4 weeks), the muscle tested (hamstrings, quadriceps, triceps surae, and the rest of the muscles), and the study design (RCT vs CT).

**Table 3:** Statistics of the subgroup analysis

Subgroup	Number of measures	Std diff in means (95% CI)		*p* Value	*Q* statistics
Type of study					
CT	3	1.822	(− 0.495 to 4.139)	0.123	
RCT	8	0.73	(0.443 to 1.017)	< 0.001^b^	
*Overall*	*11*	*0.746*	*(0.462 to 1.031)*	< *0.001*	(*Q* = 0.840; *df* (*Q*) = 1; *p* = 0.359)
Intervention duration					
≤ 4 weeks	3	0.253	(− 0.252 to 0.757)	0.326	
> 4 weeks	8	1.084	(0.428 to 1.740)	0.001^b^	
*Overall*	*11*	*0.562*	*(0.162 to 0.962)*	*0.006*	(*Q* = 3.880; *df* (*Q*) = 1; *p* = 0.049)^a^
Muscle tested					
Hamstrings	8	0.645	(0.319 to 0.971)	< 0.001^b^	
Quadriceps	5	0.425	(0.033 to 0.818)	0.034^b^	
Triceps surae	3	− 0.024	(− 0.763 to 0.714)	0.949	
Rest of the muscles^c^	3	2.864	(0.826 to 4.903)	0.006^b^	
*Overall*	*19*	*0.527*	*(− 0.150 to 0.009)*	< *0.001*^*b*^	(*Q* = 7.952; *df* (*Q*) = 3; *p* = 0.047)^a^

Positive values of Std diff in means indicates a favorable effect for foam rolling (and vice versa) on range of motion

*CI c*onfidence interval, *CT* controlled trial, *RCT* randomized controlled trial, *Std diff* standardized difference

^a^Significant difference between groups

^b^Significant difference within a group

^c^Muscles with < 3 studies were summarized to one group (i.e., Rectus femoris, Infraspinatus, Adductors)

Q statistics of the subgroup analyses revealed no significant differences between study designs (RCTs vs CTs).

Subgroup analysis showed that when the muscle group was considered there was a significant difference (*p* = 0.047) between muscle groups (hamstrings vs quadriceps vs triceps surae versus rest of the muscles). When foam roller was applied to hamstrings, quadriceps, and the rest of the muscles, it was effective in increasing ROM in the experimental compared to the control group (*p* < 0.001, *p* = 0.034, and *p* = 0.006, respectively). However, when foam roller was applied to triceps surae muscle no difference was observed in dorsiflexion (*p* = 0.949).

Moreover, subgroup analysis of the intervention duration indicated significant differences between interventions lasting ≤ 4 weeks compared to > 4 weeks (*p* = 0.049). Whilst studies lasting ≤ 4 weeks showed no significant difference in ROM improvements between the foam rolling and the control condition (*p* = 0.326), studies > 4 weeks of training showed significant ROM increases in the experimental compared to the control groups (*p* = 0.001).

## Discussion

The purpose of this review was to assess if foam-rolling training interventions can increase joint ROM in healthy participants. The main meta-analysis, which included a total of 11 studies, 46 effect sizes, and 290 participants (23.9 ± 6.3 years) revealed an increase in joint ROM with a medium magnitude of change (ES = 0.823; *p* = 0.001) in the experimental compared to the control groups. Subgroup analyses showed no significant difference between study designs (RCTs vs CTs). In contrast, when the muscles examined in the eligible studies were considered, significant increases in ROM were found when foam roller was applied on quadriceps and hamstrings but not on triceps surae. Furthermore, it was found that foam-rolling interventions longer than 4 weeks are needed to induce significant increases in joint ROM.

A recent meta-analysis [12] showed that a single foam roller treatment can acutely increase joint ROM with a medium effect size (ES = 0.74; *p* < 0.001). Along this line, in this meta-analysis it was found that foam roller training interventions longer than 4 weeks demonstrate similar ROM increments (ES = 0.823). Furthermore, Wilke et al. [12] found a small effect size for ROM increases following triceps surae treatment (ES = 0.43; *p* < 0.05), but a moderate effect for hamstrings (ES = 1.0; *p* < 0.05) and quadriceps (ES = 0.83; *p* > 0.05). This was partly confirmed by our results indicating small (ES = 0.425; *p* < 0.05) and moderate effect sizes (ES = 0.645; *p* < 0.05) for quadriceps and hamstrings, respectively. In contrast, our analyses for the triceps surae muscle showed a non-significant trivial effect size in ankle dorsiflexion following several weeks of foam rolling (ES =  − 0.024). Since the triceps surae is more distal compared to the quadriceps or the hamstrings, it is likely that less pressure can be applied on the muscle during a foam-rolling exercise compared to more proximal muscle groups. This might explain the difference found in this meta-analysis in the effect sizes between triceps surae and other major lower limb muscles (i.e., hamstrings, quadriceps) and also in the study of Wilke et al. [12]. The effect of the pressure applied on the triceps surae during foam rolling should therefore also be addressed in future studies by an additional load as suggested by Starrett and Cordoza [36]. A further explanation for the lack of changes in ankle ROM when foam rolling is applied on triceps surae muscle might be the relatively short duration training sessions used in the eligible studies. In the three studies examining triceps surae muscle, the participants were asked to foam roll their calves for 20 or 40 s [37], 3 × 20 s [38], and 3 × 30 s [27]. As a significant increase in ankle ROM was found only by Kiyono et al. [27] when 90 s of foam roller was used three times per week over 5 weeks, an association between intervention duration and ROM increases is possible. Moreover, it should be noted that the three studies that applied foam rolling of the calves have used different ankle ROM assessments. Whilst Kiyono et al. [27] performed a dynamometry measurement for assessing ankle ROM, Guillot et al. [37] used a weight-bearing lunge test and the participants of Stovern et al. [38] performed an active dorsiflexion in a sitting position. Hence, this variability of the research designs combined with the limited number of studies could have likely contributed to the trivial effect size in ankle ROM found in our meta-analysis. In addition, since the ankle joint has a much more limited ROM than the hip or knee due to bone and ligament structures [39, 40] this could possibly limit the potential for long-term increases in ankle joint flexibility. Moreover, the human triceps surae muscle tendon unit comprises muscles with short muscle fascicles and long tendinous tissues extending from the calcaneus insertion to the most distal part of the soleus [41]. Higher values in Achilles tendon stiffness [42] have been reported compared with values obtained for the human patellar tendon [43, 44] and this may also limit ROM increases following a long-term intervention. The combination and interaction of these aforementioned structural characteristics of the ankle and the triceps surae muscle tendon unit possibly explain the lack of significant increases in ankle ROM following a long-term foam-rolling intervention. The acute increase in ankle ROM following a single bout of foam rolling [12] is likely triggered by changes in pain perception [13] rather than structural changes. Moreover, it was assumed that the study duration could play an important role for long-term joint ROM increases. The subgroup analysis for study duration showed a significant difference on joint ROM between > 4 weeks’ intervention time and studies of ≤ 4 weeks (*p* = 0.049). However, when performing subgroup analyses between ≤ 5 weeks and > 5 weeks or between ≤ 6 weeks and > 6 weeks’ intervention time no such difference (5 weeks cut-off: *Q* = 0.000; *df* (*Q*) = 1; *p* = 0.996; 6 weeks cut-off: *Q* = 0.450; *df* (*Q*) = 1; *p* = 0.503) was found. Hence, we believe that 4 weeks was the right cut-off point for the studies eligible for this review. Consequently, ROM adaptations following foam rolling should exceed 4 weeks of training and practitioners as well as future researchers should take this threshold into account (see also Table 3).

Finally, subgroup analysis comparing RCTs and CTs revealed no significant differences between study designs (*p* = 0.359). However, we would like to report possible limitations in our meta-analysis due to the inclusion of various studies with different study designs. For example, LeGal et al. [45] used a repeated measures design where the participants served as their own controls (throughout a 5-weeks period) prior to the 5-weeks intervention. Hence, this led to a lower variance in the ROM parameters compared to regular CTs (with different participants as controls) or RCTs. Consequently, the effect size of LeGal et al. [45] was by far the highest with 5.7, and led to a potential risk of publication bias (Fig. 2). However, even when we excluded this study, the overall result of the meta-analysis was similar despite the lower effect size in absolute value but not in the magnitude (ES = 0.606; *Z* = 4.680; 95% CI 0.35–0.859; *p* < 0.001; *I*^2^ = 0.00). Moreover, one study applied a unilateral design with the contralateral leg as control following 3 weeks of foam roller training [46]. Although, there are studies reporting contralateral effects following a single bout of foam rolling [47, 48], to date it is not known if long-term foam-rolling interventions can induce crossover effects. According to recent unilateral study designs with longer-term static stretching training interventions, an increase in joint ROM of the contralateral leg has been observed following 12 and 24 weeks of training [24, 25]. Increased ROM of the stretched limb has been attributed to musculotendinous and neural responses [2, 15]. Stretch-induced musculotendinous changes with the stretched limb can include an increase in muscle compliance [49, 50], viscoelastic tissue changes [51], and muscle architectural adaptations [52, 53]. However, as the contralateral limb in the included studies was not mechanically stretched, musculotendinous mechanisms cannot be assumed. Therefore, it is possible that a CNS-mediated effect induced by unilateral foam rolling could affect the ROM of the contralateral, non-stretched muscle [54]. Assuming such a contralateral effect in the control leg in the study of Sandrey et al. [46], the inclusion of this study makes the results of the current meta-analysis even more robust. However, future RCTs (i.e., unilateral treatment in the intervention group vs control group without intervention) should investigate the chronic contralateral effect of foam rolling.

**Fig. 4:**
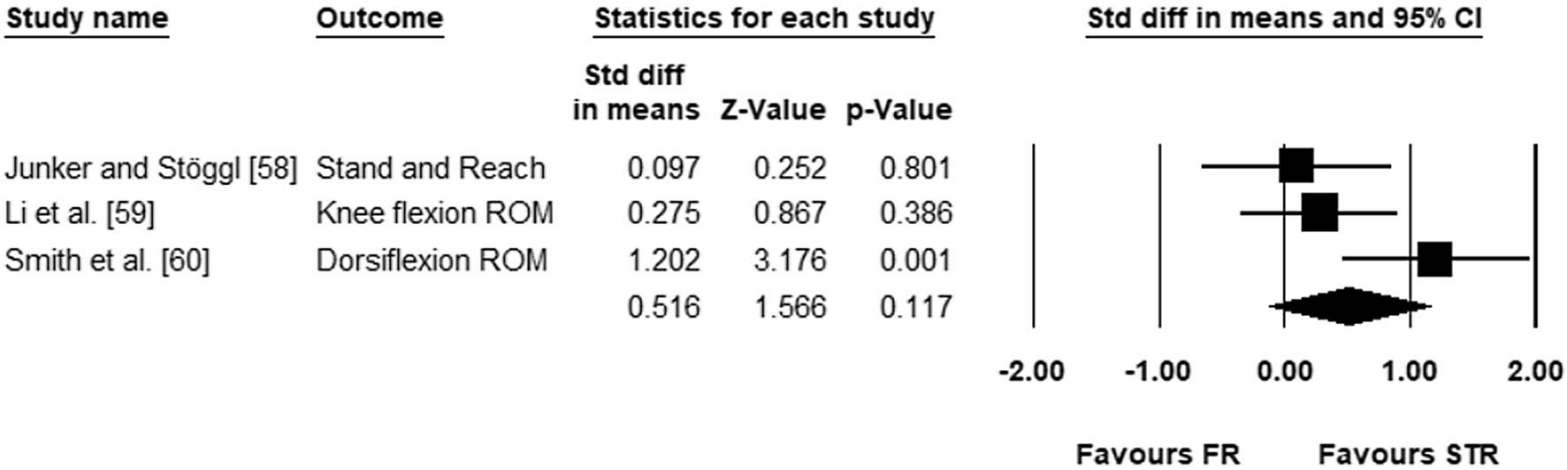
Forest plot comparing the effects of FR and STR on ROM *CI* confidence interval, *FR* foam rolling, *ROM* range of motion, *Std diff* standardized difference, *STR* stretching

Whilst the effects of a long-term stretching intervention on ROM have been comprehensively investigated during the past two decades [19–25], only 11 studies dealing with long-term training effects of foam rolling on ROM could be detected for this meta-analysis. According to our analysis, longer-term foam rolling leads to a chronic increase of ROM. Hence, it would be interesting to compare the magnitude in the increase of ROM between foam rolling and stretching. Apart from the two already included studies in the main analysis [55, 56], we have also found a further study in our systematic search [57] where the effects of foam rolling with either static stretching [56, 57], or PNF stretching [58] were compared. Our analysis showed that based on these three studies no significant difference between stretching and foam rolling exists (Fig. 4; [ES = 0.516; *Z* = 1.566; 95% CI − 0.130 to 1.161; *p* = 0.12; *I*^2^ = 60.25]). However, caution must be taken when interpreting this result since only three studies compared these two modalities (stretching vs foam rolling). Interestingly, the individual results of Smith et al. [57] showed a significant positive effect of stretching compared to foam rolling when applied on triceps surae. As mentioned above, our subgroup analysis showed that ankle dorsiflexion was not increased after foam-rolling intervention in triceps surae. Hence, according to this evidence, static stretching may be more effective than foam rolling in increasing ankle ROM when applied to triceps surae. Future studies should consider different intermuscular responses to foam rolling and stretching.

Furthermore, it would be important to investigate which mechanism causes an increase in ROM following a foam-rolling training intervention. There was only one of the 11 studies that assessed neurological (tolerance to stretch) but also structural (muscle stiffness) parameters to identify possible mechanisms [27]. Foam rolling is a type of soft tissue self-massaging that aims to release the soft tissue from the traction exerted by a fascia that has become either inelastic or adherent to adjacent tissues due to injury or pathology [59, 60]. Although it is not clear if foam rolling releases myofascia [10], acute increases in soft tissue elasticity, pain threshold, and subsequently stretch tolerance have also been observed [13] and it is assumed that altered pain perception is also a possible mechanism for long-term increases in ROM rather than changes in muscle stiffness [27]. Furthermore, such neurological changes may be caused by the friction-induced increases in temperature of the skin, muscle tissue, and fascia as well as the stress generated by the pressure exerted by rolling the muscles [61].

Regarding the various possibilities to roll a muscle in terms of frequency or in terms of pressure, little information is reported in the included studies. Only 4 of the 11 studies reported the frequency of the foam rolling application (i.e., time that was used to roll back and forth; see Table 1). Behm et al. [11] in their clinical commentary, suggested that the optimal frequency for an acute increase in ROM is likely 2–4 s of rolling time for a single roll in one direction over the length of a body part. However, this has not been tested for longer training durations, therefore future studies should compare the effects of different rolling frequencies. Although 8 of the 11 studies reported the pressure applied on the foam roller, it is not possible to compare the results due to the various types of reporting rolling pressure (e.g., 7/10 visual analogue scale [VAS] vs pressure with little discomfort). Therefore, the authors encourage use of both VAS and objective measures (force assessed on a force plate) in future studies, which would allow a better comparison of the results between the studies.

Since a single foam-rolling treatment with an additional vibration stimulus has the potential to induce positive results in terms of ROM [12, 62] and performance parameters [17, 63], future studies on the long-term effects of foam rolling should take vibration foam rolling into account. Moreover, future studies should test performance parameters (i.e., strength) to detect possible changes due to a long-term foam rolling intervention as seen following a stretching stimulus [25]. There is some evidence of performance increments following acute foam-roller interventions [64] and 5 of the 11 studies included in this meta-analysis reported strength-based measurements and particularly promising results following foam rolling [26, 38, 45, 58, 65].

This meta-analysis has some limitations. First, only three moderating variables (i.e., weeks of intervention, muscles tested, and study design) were considered for subgroup analyses. This potentially obscures further potential variables (sex, activity level, rolling intensity, rolling frequency), which might have explained the increase in ROM following a foam-rolling training intervention. Second, a moderate to high heterogeneity was found in the main meta-analysis (*I*^2^ = 72.76). This can be likely explained by, for example, varying outcome measures, participants, or intervention duration. However, the most likely explanation was the study of LeGal et al. [45] with by far the highest effect size of 5.7. Conducting the meta-analysis without LeGal et al. [45] would lead to similar results but low heterogeneity (ES = 0.606; *Z* = 4.680; 95% CI 0.35–0.859; *p* < 0.001; *I*^2^ = 0.00). Third, the conclusions drawn from our results are mainly based on a young adult population (23.9 ± 6.3 years). Hence, future studies should also investigate younger and older populations.

## Conclusion

In conclusion, our meta-analysis showed that long-term foam-rolling interventions can increase joint ROM in young healthy participants. However, ROM increases may be muscle-and/or joint-dependent as foam rolling on the triceps surae muscle did not increase ankle dorsiflexion. Moreover, our results indicate that an intervention of more than four weeks is needed to observe significant changes in ROM. Future studies should investigate the effects of a high-volume foam-rolling intervention, the effects of a vibration foam-rolling intervention, the contralateral effects of foam rolling, the possible differences in intramuscular responses (e.g., calf vs quadriceps rolling), strength-based effects (e.g., maximum torque values), and the mechanism underpinning increases in ROM. Moreover, a quantification of the pressure applied on the foam roller (i.e., with force plates) and also different frequencies of the foam-rolling application (e.g., 1 s per roll from distal to proximal vs 4 s per roll) should be examined in order to obtain a clearer picture of the long-term effects of foam rolling.

## Declarations

### Availability of Data and Material

All data will be made available on request to the corresponding author.
